# Infantile Hypoxic Encephalopathy Mimicking Acute Encephalopathy with Biphasic Seizures and Late Reduced Diffusion (AESD) Identified as an Episode of Brief Resolved Unexplained Event (BRUE)

**DOI:** 10.3390/jcm12165239

**Published:** 2023-08-11

**Authors:** Shuhei Fujino, Mikako Enokizono, Tatsuo Kono, Sahoko Miyama

**Affiliations:** 1 Department of Neurology, Tokyo Metropolitan Children’s Medical Center, Tokyo 183-8561, Japan; sahoko_miyama@tmhp.jp; 2 Department of Radiology, Tokyo Metropolitan Children’s Medical Center, Tokyo 183-8561, Japan; mikako_enokizono@tmhp.jp (M.E.); tatsuo_kono@tmhp.jp (T.K.)

**Keywords:** acute encephalopathy with biphasic seizures and late reduced diffusion, hypoxic encephalopathy, brief resolved unexplained event, bright tree appearance, glutamate excitotoxicity, nonconvulsive status epilepticus

## Abstract

Acute encephalopathy with biphasic seizures and reduced diffusion (AESD) is characterized by biphasic seizures following febrile viral infections and delayed reduced diffusion of the cerebral white matter on magnetic resonance imaging (MRI) diffusion-weighted imaging (DWI) (bright tree appearance, BTA). However, hypoxic encephalopathy with biphasic seizures and AESD-mimicking imaging findings has not been reported. We report a case of hypoxic encephalopathy due to suffocation with concomitant biphasic seizures and BTA, mimicking AESD. On day 1, a healthy 5-month-old girl was found face down with decreased breathing and a deteriorating consciousness level, suggesting a brief resolved unexplained event (BRUE). Electroencephalography (EEG) revealed periodic epileptic discharges, suggesting possible nonconvulsive status epilepticus. Despite improvements in consciousness level and EEG abnormalities on day 2, her consciousness level deteriorated again with generalized tonic–clonic seizures on day 3, and a head MRI-DWI revealed restricted diffusion predominantly in the subcortical areas, suggesting BTA. Treatment for acute encephalopathy resolved the clinical seizures and EEG abnormalities. Persistence of abnormal EEG, reflecting abnormal excitation and accumulation of neurotoxic substances caused by hypoxia, may have contributed to the development of AESD-like findings. As hypoxic encephalopathy causes AESD-like biphasic seizures, monitoring consciousness level, seizure occurrence, and EEG abnormalities even after acute symptoms have temporarily improved following hypoxia is essential.

## 1. Introduction

Several hundred cases of acute encephalopathy with biphasic seizures and late reduced diffusion (AESD), a common form of acute encephalopathy among Asian children, are recorded each year [1,2]. AESD is characterized by biphasic seizures and delayed reduced diffusion several days after the onset, mainly in the frontal and occipital subcortical white matter on MRI diffusion-weighted imaging (DWI), which is referred to as the bright tree appearance (BTA) with central sparing [1]. In AESD, early-phase seizures develop during febrile viral infections, whereas late-phase seizures typically occur 3–7 days after temporary improvement [1]. The pathogenesis of AESD is thought to be glutamate excitotoxicity resulting from seizures in the first phase of status epilepticus [3,4]. Because AESD is associated with severe neurological sequelae, seizures should be monitored carefully and may be treated early to suppress excitotoxicity [1]. However, there have been no reports of hypoxic encephalopathy with biphasic seizures and imaging findings mimicking AESD. In patients with hypoxic encephalopathy, neurological symptoms, including seizures, are generally monophasic.

We present a case of hypoxic encephalopathy caused by suffocation, which was identified as an episode of brief resolved unexplained event (BRUE), leading to the development of biphasic seizures and the detection of BTA-like findings. BRUE is defined as a brief, sudden event that occurs in children <1 year of age with no apparent background illness who meet one or more of the following criteria: (1) cyanosis or pallor; (2) absent, decreased, or irregular breathing; (3) marked change in tone (hyper- or hypotonia); and (4) altered level of responsiveness. The concept previously included apparent life-threatening event (ALTE) or near-miss sudden infant death syndrome (SIDS). Although BRUE is diagnosed when there is no apparent background illness to explain the event, it may sometimes be associated with various potential causes such as child abuse, gastroesophageal reflux, epilepsy, central nervous system disease, arrhythmia, cardiomyopathy, respiratory tract infection, sepsis, meningitis, suffocation, airway obstruction, or metabolic abnormalities [5].

Hypoxic encephalopathy is caused by hypoxia and/or reduced cerebral blood flow (ischemia), resulting in hypoxic brain tissue. In children, hypoxemia is more often a consequence of asphyxial events and related to suffocation, dehydration, neonatal anoxia, and child abuse, whereas in adults brain hypoxia/ischemia is usually secondary to cardiopulmonary arrest or cerebrovascular disease [6,7]. However, pure hypoxia is uncommon in clinical practice, and distinguishing hypoxia from hypoxia–ischemia is difficult because prolonged hypoxemia leads to cardiac hypoxia and a rapid decrease in cardiac output within a few minutes [6]. In the acute phase, increased anaerobic glycolysis due to hypoxia can elevate the lactate level and cause ketosis. Tissue hypoxia and hypoperfusion of the systemic organs can also result in elevated creatine kinase (CK) levels and the deviation of liver enzymes [8]. The watershed region between the distributions of the cerebral arteries is vulnerable to hypoxia [6]. The occipitotemporal cortex, basal ganglia, and hippocampi are more vulnerable to hypoxic injury than other brain areas. Bilateral and symmetric damage occurs almost invariably [6]. Brain computed tomography (CT) in the acute phase shows nonspecific signs such as cerebral edema and indistinct corticomedullary borders [6]. In the acute phase, lesions are better depicted using DWI because of the presence of cytotoxic edema. Lactate peaks may be observed on magnetic resonance (MR) spectroscopy [6]. During the late subacute and chronic phases, atrophic changes predominate the affected areas, resulting in the dilatation of cerebrospinal fluid spaces. Cortical laminar necrosis is also seen during these stages, because cortical layers 3, 5, and 6 are relatively vulnerable to hypoxia [6,9].

Herein, we report an infantile case of hypoxic encephalopathy combined with biphasic seizures and BTA-like findings that mimicked the clinical course of AESD.

## 2. Case Report

### 2.1. Patient Presentation

A 5-month-old female patient who seemed to have suffocated was found lying prone on a cushion by her guardians. When the patient was discovered, she was pale, her breathing rate was decreased, and her responsiveness was altered. Her guardians immediately called an ambulance. Emergency staff administered oxygen using a bag-valve mask. Cardiopulmonary resuscitation (CPR) was not performed as her heart rate remained above 100 beats/min. She had previously been healthy, with no history of perinatal or developmental abnormalities. She had no feeding problems or growth retardation. There was no history of epilepsy or family history of sudden death. There was no history of vomiting or gastroesophageal reflux (GER), and no vomit marks were found around her at the time of discovery. Neonatal screening for metabolic diseases was negative. Her parents reported that she had recently developed the capacity to roll over by herself. On the day of onset, she had no symptoms of respiratory tract infection or gastroenteritis.

### 2.2. Physical Findings

**Figure 1:**
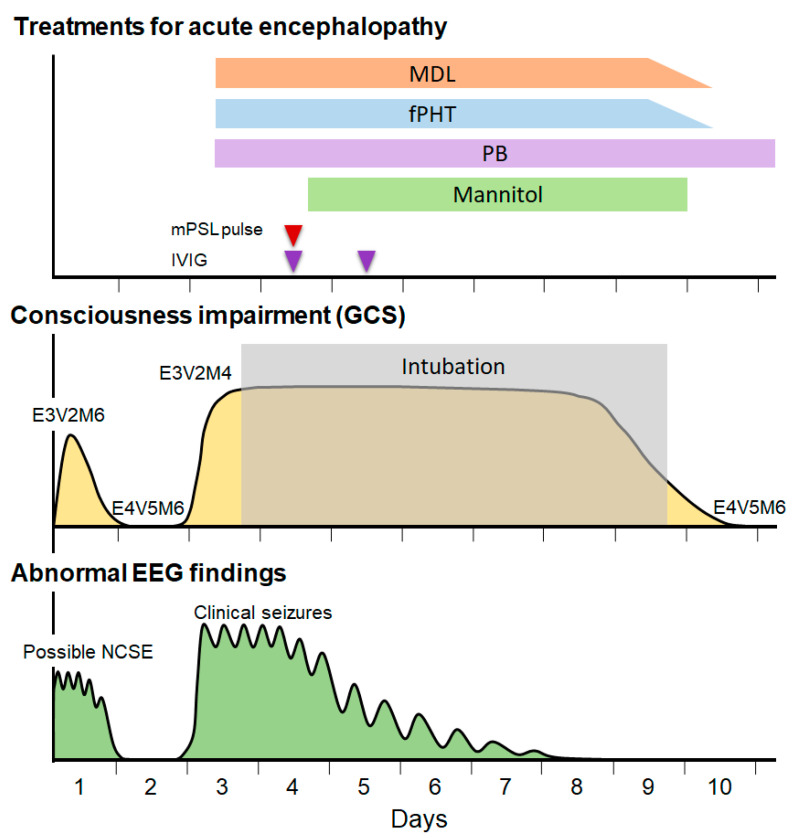
The patient’s biphasic clinical course and treatment in the acute period. Abnormalities of electroencephalogram (EEG), seizures, and impairment of consciousness showed a biphasic course. MDL, midazolam; fPHT, fosphenytoin; PB, phenobarbital; mPSL, methylprednisolone; IVIG, intravenous immunoglobulin; GCS, Glasgow Coma Scale.

When the patient arrived in our emergency department, her vital signs showed a heart rate of 170 beats/min, respiratory rate of 60/min, and blood pressure of 110/76 mmHg. The patient’s consciousness level was E3V2M4 on the Glasgow Coma Scale (GCS) and her body temperature was 37.0 °C. Eye opening was observed in response to mild pain or sound stimuli but her eyes were misaligned (Figure 1). There was no evidence of retinal hemorrhage. There was no hepatomegaly. No signs of trauma were evident on the body’s surface or on whole-body skeletal radiography.

### 2.3. Laboratory and Imaging Findings upon Admission

**Figure 2:**
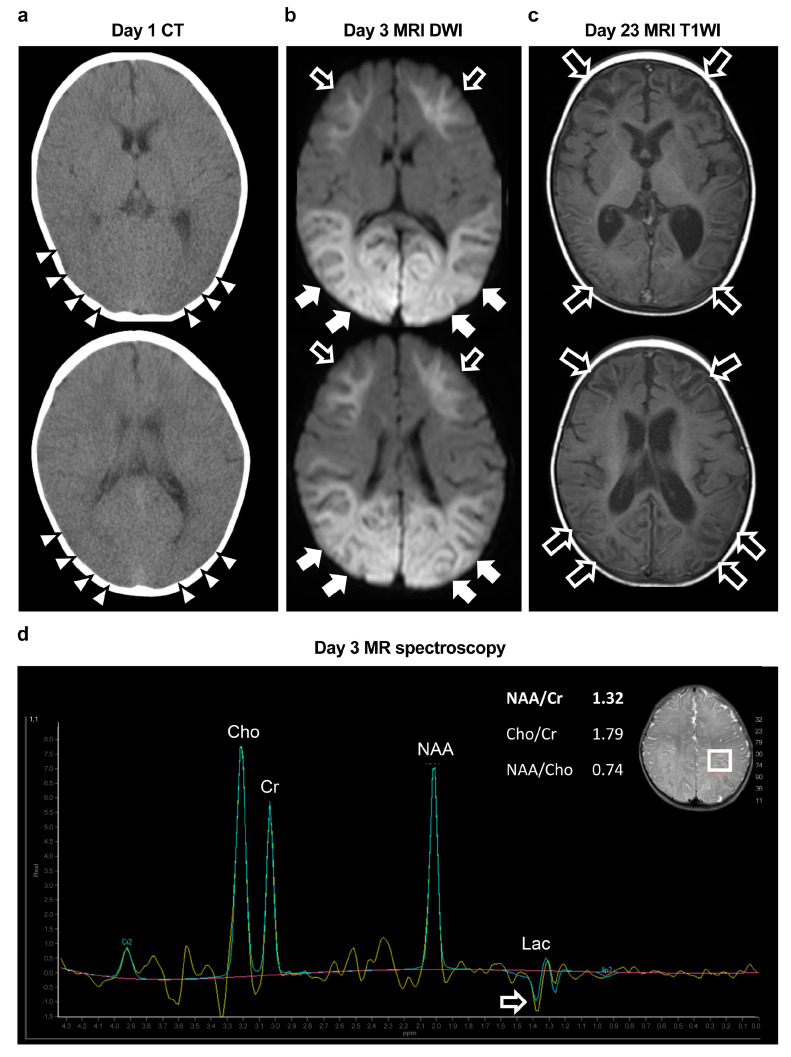
Changes in imaging findings during the acute to subacute phases. (**a**) Head CT (Day 1): Diffuse cerebral edema and indistinct corticomedullary borders (white arrowhead). (**b**) Head MRI-DWI (Day 3): High-intensity lesions can be seen extending throughout the subcortical white matter and deep white matter of the frontal lobes (bright tree appearance, black arrows). In the occipital and lateral temporal lobes, the high signal extends to the cortex (white arrows). No high-intensity area was observed around the central cerebral sulcus (central sparing). (**c**) Head MRI T1-weighted imaging (T1WI) during the subacute period (day 23). Curvilinear hyperintense lesions were observed mainly in the cortex of the frontal and occipital lobes (cortical laminar necrosis, black arrows). Significantly decreased volumes of the cerebral cortex hemispheres, basal ganglia, hippocampi, and cerebral corpus callosum were also observed. (**d**) Proton MR spectroscopy of the parietal lobe (white square, volume of interest, 15 × 20 × 20 mm^3^; echo time [TE] = 144 msec; repetition time [TR] = 2000 msec) on day 3. No specific spectrum was observed, except for a slightly inverted lactate peak (black arrow). Lac, lactate; NAA, N-acetylaspartate; Cr, creatine; Cho, choline.

Chest X-ray showed no abnormalities in the lung fields or cardiac shadows. Electrocardiogram was regular and showed no abnormal findings such as QT prolongation. Blood biochemistry revealed elevated levels of aspartate aminotransferase (AST 263 U/L), alanine transaminase (ALT 76 U/L), lactate dehydrogenase (LDH; 976 U/L), CK (9005 U/L), and serum lactate (13.8 mmol/L). C-reactive protein level was normal. The patient’s complete blood count showed an elevated white blood cell count of 17,090/µL; however, other parameters were normal, and the hemoglobin level was 11.5 g/dL. Arterial blood gas analysis (ABG) revealed a pH of 7.105, PaCO_2_ of 25.6 mmHg, PaO_2_ of 72.6 mmHg, HCO_3_^−^ of 9.5 mmol/L, base excess (BE) of −20.0 mmol/L, and an anion gap (AG) of 16.8 mmol/L. Her blood glucose level was 364 mg/dL at the time of emergency transport, with no evidence of hypoglycemia. Blood amino acid analysis showed no abnormalities. Urinary organic acid analysis showed elevated lactate peaks. Serum levels of total ketones bodies, 2-OH butyric acid, and acetoacetic acid were 1224 µmol/L, 876 µmol/L, and 348 µmol/L, respectively. Urine ketone bodies were 2+. A cerebrospinal fluid (CSF) examination revealed no abnormalities. Polymerase chain reaction testing of the CSF was negative for viruses, including those that frequently cause acute encephalopathy (human herpes virus (HHV)-6, HHV-7, influenza virus, respiratory syncytial virus, rotavirus, herpes simplex virus, and enterovirus). Urine organic acid and blood amino acid analyses were unremarkable. Head CT revealed diffuse cerebral edema and an indistinct corticomedullary border (Figure 2a).

### 2.4. Clinical Course after Hospitalization

**Figure 3:**
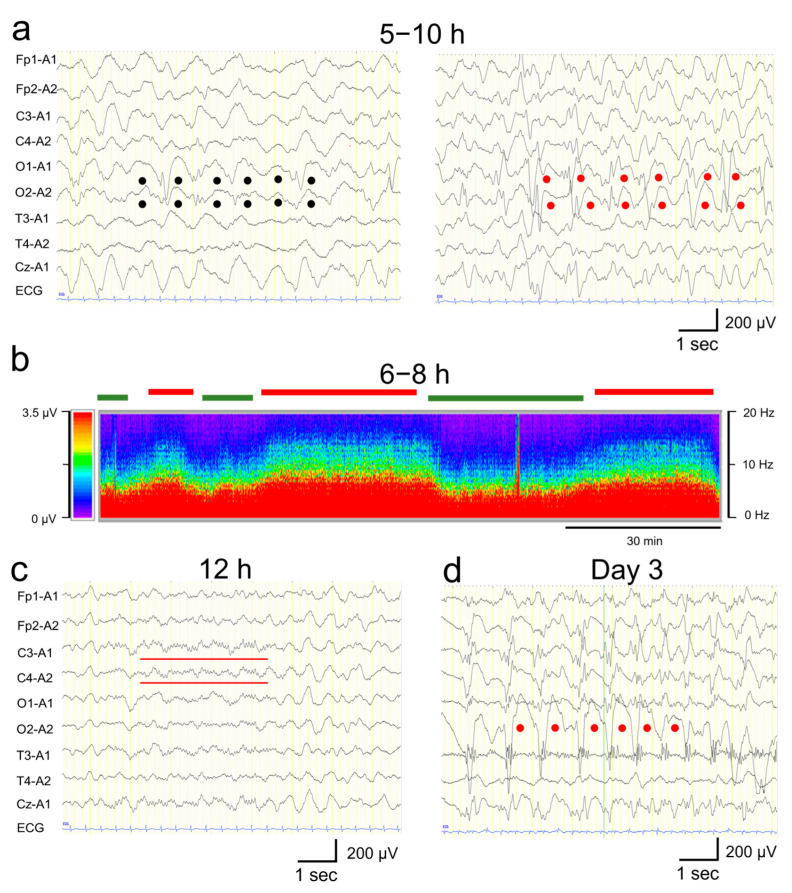
Electroencephalogram (EEG) findings. (**a**) EEG findings 9 h after the onset: High-amplitude slow waves (1–3 Hz, 150–250 μV) were observed predominantly in the occipital region (black dots, **left panel**). Rhythmic 1–3 Hz, 200–350µV spike-and-wave complexes including 6–10 Hz, 200–350 µV spikes were observed predominantly in the bilateral occipital region (red dots, **right panel**). (**b**) Density-modulated spectral array obtained from EEG waveforms during the first 6–8 h from onset. The vertical axis indicates the frequency (in the 0–20 Hz range), the horizontal axis indicates the time, and the color change indicates the amplitude intensity based on the EEG frequency spectrum using the Fast Fourier Transform analysis (in the 0–3.5 µV range). A phase containing 1–3 Hz spike-and-wave components and 6–10 Hz spike components (red lines on top) and a phase consisting of <5 Hz slow waves (green lines) alternate every 15–30 min. (**c**) EEG findings 12 h after the onset. The high-amplitude slow waves and spikes disappeared, and the amplitude decreased throughout the region. Spindle-like fast waves were also observed partially in C3 and C4 (red bars). (**d**) EEG findings on day 3. Right, occipitally predominant, rhythmic spikes, slow waves, and spike-and-wave complexes were frequently observed (red dots). Monopolar recordings: sensitivity, 10 mV; high-cut filter, 30 Hz; alternating current filter, off; time constant, 0.1 s.

The patient was managed in the intensive care unit after admission. ABG analysis after admission indicated a pH of 7.398, PaO_2_ of 193 mmHg, PaCO_2_ of 33 mmHg, BE of −3.7 mmol/L, HCO_3_^−^ of 21.3 mmol/L, AG of 11.2 mmol/L, and serum lactate level of 1.8 mmol/L, indicating rapid improvement in metabolic acidemia and hyperlactatemia. Her blood glucose level was normalized to 110 mg/dL without the administration of hypoglycemic agents, and CK peaked on day 1 and rapidly declined to 214 mg/dL by day 5. The level of urine ketone bodies became negative on day 2. Continuous EEG monitoring was started after admission. From 5 to 10 h after the onset, a phase consisting of 1–3 Hz, 150–250 µV slow wave bursts (Figure 3a, left panel), and another phase composed of 1–3 Hz, 200–350 µV rhythmic (poly)spike-and-wave complexes including 6–10 Hz, 200–350 µV spike component (Figure 3a, right panel) were observed predominantly in the bilateral occipital region without apparent seizures (Figure 3a,b). These phases alternated every 15–30 min (Figure 3b). Based on the fluctuation in the EEG findings and the patient’s impaired consciousness, a diagnosis of “possible NCSE” was made [10]. No antiepileptic drugs were administered at this time, as the patient’s level of consciousness and EEG findings were improving by 12 h after the onset (Figure 1). Twelve hours after the onset, her consciousness level improved to E4V5M6 on the GCS, and the abnormal waves resolved with the appearance of spindle-like fast waves on the EEG (Figure 1 and Figure 3c). Mechanical ventilation was not required until day 3.

On day 3, however, the patient’s consciousness level deteriorated to E3V2M4 (GCS), and she experienced a cluster of generalized tonic–clonic seizures. EEG revealed diffuse spike-and-wave complexes predominantly in the occipital region (Figure 3d). Head MRI-DWI on the same day showed reduced diffusion in the subcortical areas, except the perirolandic regions invoking BTA with central sparing (Figure 2b). In the occipital and lateral temporal lobes, DWI high signal areas extended into the cortex as well as the subcortex (Figure 2b). MR spectroscopy (echo time [TE] = 144 msec, repetition time [TR] = 2000 msec) revealed only a slightly inverted lactate peak (Figure 2d).

### 2.5. Diagnosis, Treatment, and Outcomes

Based on the biphasic course that is unusual for hypoxic encephalopathy, a diagnosis of secondary AESD-like acute encephalopathy due to hypoxic encephalopathy was made. On day 3, treatment was initiated with artificial ventilation; methylprednisolone pulse therapy (30 mg/kg/day); intravenous immunoglobulin therapy (1 g/kg/day); administration of D-mannitol (up to 4 g/kg/day); and intravenous anticonvulsant therapy with midazolam (MDL), phenobarbital (PB), and fosphenytoin (fPHT) immediately after the emergence of second seizures (Figure 1). To suppress acute clinical seizures, we used a MDL dose of up to 6 mg/kg/day, PB of up to 40 mg/kg/day, and fPHT of 7.5 mg/kg/day following an initial dose of 22.5 mg/kg/day. As the clinical seizures and abnormal EEG resolved and her consciousness level improved to E4V5M6 on the GCS by day 10, these treatments were discontinued (Figure 1). Head MRI T2-weighted imaging on day 23 revealed laminar necrosis in the frontal and occipital cortices, thinning of the corpus callosum, and a decrease in the volume of the bilateral cerebral hemispheres, basal ganglia, and hippocampi (Figure 2c). After that, she did not have any abnormalities in her respiratory rhythm. One year after onset, at the age of 1.5 years, she experienced visual cognitive dysfunction but could utter ten meaningful words and walk without assistance.

Written informed consent was obtained from the patient’s parents for the publication of this case report. A Neurofax EEG-1250 (Nihon Kohden, #code: EEG-1250) was used for EEG measurement and analysis.

## 3. Discussion

The biphasic clinical course and reduced diffusion predominantly in the subcortical white matter on the head MRI in the present patient indicated an AESD-like clinical course. However, there was no evidence of a viral infection or febrile period, which is almost invariably observed in AESD.

This patient presented with BRUE that resolved spontaneously without apparent resuscitation. BRUE is defined by the absence of an obvious causative disease; however, the associated causes, such as GER, epilepsy, respiratory tract infection, asphyxia, arrythmia, or metabolic diseases, can be detected [5]. In this case, there was no history suggestive of GER, such as vomiting or choking, and there were no traces of vomiting or images of aspiration pneumonia at the time of discovery. The absence of any signs of infection, including upper or lower respiratory infection, is the notable feature of this case. Electrocardiogram and chest X-ray findings were normal and cardiogenic diseases were also ruled out, as there was no family history of arrhythmia or sudden death. The differential diagnosis of hypoxic encephalopathy comprises various conditions including hypoglycemia, anemia, mitochondrial diseases, and carbon monoxide poisoning [6]. Although the spatial distribution of hypoglycemic brain lesions is similar to that observed in hypoxic encephalopathy and it significantly affects the parietal and occipital regions and the hippocampus, no hypoglycemia was observed in this case. This case showed no significant anemia. Lactic acidemia with raised AG is seen in patients with mitochondrial disease, congenital metabolic diseases (e.g., glycogen storage disorders, disorders of gluconeogenesis, and pyruvate dehydrogenase deficiency) and hypoxia [11]; however, this infant did not have hypoglycemia, abnormalities on neonatal metabolic screening, blood amino acid analysis, or any clinical symptoms before the onset. There was also no obvious history of carbon monoxide poisoning. Epilepsy or near-miss sudden unexpected death in epilepsy (SUDEP) is one of the differential diagnoses of BRUE. The risks of SUDEP include being an adolescent and having epilepsy for more than 5 years [5]; thus, the occurrence of near-miss SUDEP was unlikely in this case, considering the patient was a healthy infant with no history of epilepsy and no family history of sudden death or epilepsy. Even if epilepsy was present at the onset, the cause of lactic acidemia and ketosis cannot be explained. In addition, the absence of obvious epileptic discharge on EEG at the time of hospital admission suggests that the seizures did not sustain in this case. Upon arriving at the emergency department, the patient’s heart rate was >100 beats/min and no CPR was performed; thus, post-resuscitation encephalopathy is unlikely. However, it should be noted that both hypoxic encephalopathy and post-resuscitation encephalopathy can be caused by hypoxic ischemia in the brain, and imaging findings may not distinguish between the two.

Having ruled out a differential disease as the cause of BRUE, we further present six pieces of evidence of the initial presence of hypoxic encephalopathy.

1. Based on her present history, sleeping in a prone position on a cushion was believed to be the cause of suffocation. Lying prone on soft bedding may have caused the elevation of her diaphragm, CO_2_ rebreathing, and frequent oxygen desaturation due to airway obstruction [12]. However, because the circumstances under which the guardian found the child remain unknown, it is necessary to be cautious in diagnosing this as a case of asphyxia caused by a prone sleeping position based on the recorded medical history alone. In other words, it is necessary to differentiate other diseases from the patient’s medical history and examination findings as mentioned above;
2. Diffuse brain edema and indistinct corticomedullary borders developed within a few hours of onset. In excitotoxic encephalopathy, including a typical instance of AESD, abnormalities on imaging findings typically appear several days after the onset [1,7]. In addition, abnormal subcortical white matter signals extending to the occipital and lateral temporal cortex were not typical of BTA in AESD [1,3]. Therefore, we considered these AESD-like findings to have appeared because of primary hypoxic encephalopathy;
3. A significant increase in the level of serum lactate and enzyme deviation, reflecting neuronal injury and tissue hypoxia, was also observed immediately after the onset of hypoxic encephalopathy. At the time of transport to our hospital, the patient had metabolic acidemia, hyperlactatemia, ketosis, and markedly elevated levels of CK, LDH, and other deviating liver enzymes. Hyperlactatemia, metabolic acidemia, and ketosis were improved quickly after starting intensive care, allowing us to conclude that these conditions were caused by the event at the onset and occurred due to increased anaerobic metabolism caused by hypoxia [8]. As the levels of lactate and other deviating enzymes are rarely elevated immediately after the onset of excitotoxic encephalopathy including AESD [1,3,13], it is thought that the abnormally high levels of CK and deviating liver enzymes, lactic acidemia, and ketosis seen in this case may reflect severe asphyxia associated with BRUE;
4. A slight lactate peak on the MR spectroscopy was observed in our case, which indicated acute hypoxia and cerebral ischemia reflecting anaerobic brain metabolism, suggesting that hypoxia was already present at the time of onset [6];
5. Cortical laminar necrosis, which manifests as subacute, curvilinear, hyperintense cortical lesions on T1-weighted imaging, indicated selective hypoxic necrosis of cortical layers 3, 5, and 6, which are particularly vulnerable to hypoxia, as was observed in our case [6,9];
6. The reduction in the volume of the cerebral cortex, cerebral white matter, basal ganglia, hippocampi, thalamus, and corpus callosum became apparent during the subacute stage of hypoxic encephalopathy [6]. This distribution of lesions was consistent with the findings of hypoxic encephalopathy. However, it should be noted that such distribution can also occur in hypoglycemic encephalopathy and extremely severe cases of AESD [1,6,7].

Based on these findings, hypoxic encephalopathy was considered as the primary etiology in this patient.

Hypoxic encephalopathy does not always cause biphasic seizures; however, in the present case, continuous NCSE and brain damage due to hypoxia and the subsequent accumulation of neurotoxic substances might have contributed conjointly to the second-phase seizures, just as in AESD. Although the pathogenesis of AESD remains unclear, recent studies have suggested that biphasic seizures in AESD are caused by neuronal death, astrocyte dysfunction, and neuronal edema associated with excessive glutamate excitotoxicity resulting from seizures that occurred in the early phase [3,4]. In this case, there were epileptic discharges of <2.5 Hz and periodic EEG fluctuation, confirming the diagnosis of possible NCSE based on the Salzburg criteria [10]. It was suggested that the persistence of abnormal EEG reflecting abnormal excitation due to hypoxic damage in the first phase may have caused glutamate excitotoxicity. Additionally, in hypoxic encephalopathy, impaired ATP production and lactate accumulation due to hypoxia are associated with the depolarization of neuronal cells and the release of excitatory neurotransmitters, including glutamate. The overactivation of glutamate receptors causes a Ca^2+^ influx via receptor-mediated Ca^2+^ channels, which activate calcium-dependent intracellular enzymes, which, in turn, lead to the accumulation of reactive oxygen/nitrosative species and secondary brain injury 24 h after the onset of hypoxia [6,13,14]. Reactive oxygen/nitrosative species damage cell membranes and mitochondria, further impairing ATP production and causing the depletion of neuronal energy [6]. Excessive activation of glutamate receptors promotes the influx of ions such as Ca^2+^, Na^+^, and Cl^−^, causing an overload of intracellular ions and an influx of water into the cell [6]. The resulting neuronal edema affects the surrounding sites by reducing perfusion, causing secondary depolarization in the vicinity of the primary disorder that spreads the edema [6]. In the course of this progressive hypoxic and/or ischemic injury, hemodynamic, metabolic, and ionic changes heterogeneously alter the affected area, so that even initially, barely surviving neurons may enter apoptosis, or delayed, programmed cell death [6]. These phenomena may explain why brain regions rich in glutamate and other excitatory amino acid receptors, such as gray matter and regions of high metabolic demand, are more susceptible to hypoxic and/or ischemic injury [6]. They may also explain the appearance of delayed lesions after the 24 h following a hypoxic event [6].

Etiologies other than infection, such as head trauma, have been reported to produce AESD-like findings; i.e., biphasic seizures and BTA [1,7,15,16]. Takase et al. reported a patient with head trauma resulting in an acute subdural hematoma who presented with AESD-like biphasic seizures and delayed reduced diffusion, indicating BTA [17]. In their report, they stated that cytotoxic edema, microglial activation, glutamate excitotoxicity, and intracellular Ca^2+^ influx due to diffuse brain damage can cause AESD-like clinical findings [17]. In our case, just like in the trauma case, extensive brain damage due to hypoxia and neuronal excitotoxicity and increased intracellular Ca^2+^ influx may have contributed to the development of AESD-like findings.

In the present case, reduced diffusion seen on the day 3 MRI-DWI was limited to the subcortical area in the anterior lobe, invoking BTA; however, the fact that the high signals on the DWI extended to not only the subcortical area but also the cortex of the occipital and partially temporal lobes was not typical of BTA. BTA is one of the specific features of AESD that can be caused by excitotoxicity, as described above [1,3,4]. These findings are thought to reflect reduced water motion in most of the white matter, except the central sulcus regions in the anterior lobes, and lesions are typically limited to the subcortex in AESD [7]. On the other hand, in hypoxic encephalopathy, it is known that reduced water motion can be broadly observed in most of the cerebral cortex as well as the subcortical white matter [7]. It is suggested that the coexistence of typical BTA and high DWI signals extending to the cortex may have been the result of a combination of glutamate excitotoxicity, as well as hypoxic brain injury and the subsequent accumulation of neurotoxic substances in our case.

## 4. Conclusions

It is necessary for not only pediatric neurologists but also pediatric generalists to understand the concept of AESD and that hypoxic encephalopathy can result in an AESD-like biphasic clinical course with BTA-like findings. Therefore, it is necessary to monitor the patient’s consciousness level, seizure occurrence, and changes in EEG and MRI findings carefully even after the seizures and/or consciousness have temporarily improved following hypoxia.

## Data Availability

The data that support the findings of this report are available from the corresponding author, SF, upon reasonable request.
